# Finding acceptance: How adolescents and young adults with Klinefelter Syndrome navigate identity, disclosure, and support

**DOI:** 10.1002/jgc4.70074

**Published:** 2025-06-24

**Authors:** Abigail Tubman, Jaclyn L. Papadakis, Courtney Finlayson, Debra Duquette, Allison Goetsch Weisman

**Affiliations:** ^1^ Graduate Program in Genetic Counseling Northwestern University Chicago Illinois USA; ^2^ Department of Psychiatry & Behavioral Health Ann & Robert H. Lurie Children's Hospital of Chicago Chicago Illinois USA; ^3^ Department of Psychiatry & Behavioral Sciences Northwestern University Feinberg School of Medicine Chicago Illinois USA; ^4^ Department of Pediatrics Northwestern University Feinberg School of Medicine Chicago Illinois USA; ^5^ Division of Endocrinology Ann and Robert H. Lurie Children's Hospital of Chicago Chicago Illinois USA; ^6^ Department of Medicine Northwestern University Feinberg School of Medicine Chicago Illinois USA; ^7^ Division of Genetics, Genomics, and Metabolism Ann and Robert H. Lurie Children's Hospital of Chicago, Northwestern University Feinberg School of Medicine Chicago Illinois USA

**Keywords:** disclosure, identity, Klinefelter Syndrome, psychosocial well‐being, support system

## Abstract

Klinefelter syndrome (KS; 47, XXY) is the most common chromosomal aneuploidy, affecting ~1:600 male births. Due to recent technological advances, KS is diagnosed more often in young individuals. This emerging population of adolescent and young adult (AYA) patients with KS seeking care makes it increasingly important to understand the unique experiences and perspectives of these individuals. This qualitative study explored (1) the psychosocial well‐being of AYA with KS and (2) resources and support systems AYA with KS utilize to cope with challenges related to their diagnosis. This study included 13 participants ages 14–23 (*M* age 16 years) who completed semi‐structured individual interviews. Reflexive thematic analysis was used. Three themes were generated concerning psychosocial well‐being, resources, and support systems of AYA with KS: (1) “I'm a little different”—Rejecting stigmatization and integrating a diagnosis of KS into one's identity, (2) “Let it out”—Lessons learned when disclosing one's diagnosis of KS to others, and (3) “They accept me for who I am”—The building blocks of a support system. Participants largely viewed KS as part of themselves but not their whole identity and expressed not wanting to be labeled by others as “disabled.” Participants voiced that disclosing KS to trusted others often provides a sense of relief, and that it is important to have a varied support system that includes individuals like parents, friends, and romantic partners. This study improves the understanding of what support resources AYA with KS are using, or not using, and provides insights into their psychosocial well‐being, which in turn may help providers implement effective clinical interventions and promote better psychosocial outcomes for this growing patient population.

**What is known about this topic**

Existing literature has shown that potential infertility and psychological comorbidities have negative impacts on individuals with Klinefelter Syndrome (KS), however, much of this research has focused on the adult population. Increasing diagnostic rates of KS due to genetic testing and screening advances has led to a growing pediatric population of individuals with KS. There is limited information on the perspectives and experiences of these individuals, particularly adolescents and young adults (AYA) regarding the impact of KS.

**What this paper adds to the topic**

This study provides insights into the psychosocial well‐being and support systems used by AYA with KS. This study reports the importance of integrating KS into one's identity over time in addition to emphasizing the benefits of disclosing one's diagnosis of KS to trusted others. This study also shows the importance of a varied support system for AYA with KS along with the need to search for ways to actively engage these individuals in the KS community.

## 1 INTRODUCTION

Klinefelter Syndrome (KS) is the most common chromosomal aneuploidy, affecting approximately 1:600 male births and is characterized by a karyotype of 47, XXY (Bearelly & Oates, 2019; Zitzmann et al., 2020). The phenotypes of individuals with KS vary greatly without a distinct presentation; the most frequently seen characteristics include small testes, azoospermia, and hypergonadotropic hypogonadism (Aksglaede et al., 2020; Zitzmann et al., 2020). Historically, it was estimated that only ~25% of individuals with KS received a clinical diagnosis in their lifetime, with a median age of diagnosis of 27 years (Berglund et al., 2019; Bojensen et al., 2003; Chang et al., 2020). Conversely, ~50%–70% of males with KS were thought to remain undiagnosed due to the lack of associated distinct features (Abramsky & Chapple, 1997; Bearelly & Oates, 2019; Herlihy, Halliday, et al., 2011; Herlihy, McLachlan, et al., 2011). In recent years, cell free DNA (cfDNA) screening technology has increased the overall diagnosis rate in the United States for individuals with KS due to prenatal suspicion of X chromosome aneuploidy (Bearelly & Oates, 2019).

Despite an increase in the diagnostic frequency of KS, no practice guidelines exist in the United States for the care of individuals with KS. Instead, many providers rely on their past clinical experience and the current literature to guide medical management decisions (Zganjar et al., 2020). In 2020, the European Academy of Andrology was the first and only professional society to release a comprehensive guideline for the care of individuals with KS from childhood through adulthood (Zitzmann et al., 2020). The guideline addresses common medical concerns among those with KS, including hormone replacement, infertility, and fertility preservation. Additionally, the guideline acknowledges the psychological comorbidities of KS (i.e., anxiety, depression, inattention) (Fjermestad et al., 2020; Janusz et al., 2020; Skakkebaek et al., 2018; Turriff et al., 2011) and encourages providers to monitor for these, but it does not provide any further guidance.

While studies have demonstrated that potential infertility and psychological comorbidities have an adverse impact on individuals with KS (Turriff et al., 2015, 2017), most of the research to date has focused on the adult population. Among research done in the AYA population, much of it has been conducted with samples that include both KS and other differences of sex development (DSD; a group of diagnoses in which sex chromosomes, hormone levels, internal reproductive anatomy, and/or external genital appearance differ from typical binary male or female pathways of development; Hughes et al., 2006), which makes it difficult to know how findings are representative of AYA with KS. Because the uptake of cfDNA screening technology in pregnancy is leading to KS being diagnosed more frequently at younger ages, it is increasingly important to understand the unique experiences and perspectives of AYA with KS. The present study aimed to explore the perspectives of AYA with KS regarding their experiences with making meaning of their diagnosis, how they decide to disclose their diagnosis to others, and the support systems used to cope with challenges related to living with KS. Exploration of the perspectives of AYA with KS may help clinicians better understand the patient experience and how these factors contribute to psychosocial well‐being, which may lead to improvements in clinical practice.

## 2 METHODS

The study was approved by the Institutional Review Board of Ann & Robert H. Lurie Children's Hospital of Chicago (2023‐6234).

### 2.1 Participants and recruitment

Individuals ages 14–25 years old with a diagnosis of KS (at least one 47, XXY cell line) and a minimum education level of ninth grade were eligible to participate. Eligible participants were identified via a retrospective chart review of 154 patients seen between 2012 and 2023 for KS at a single pediatric institution. Of the identified patients, 54 met eligibility criteria. Potential participants or their guardians were contacted via MyChart (patient accessible medical records database) message, email, and/or in person in clinic with recruitment fliers, with a maximum of three attempted contacts. Additionally, participants were recruited through Living with XXY, a United States‐based KS support organization. This organization advertised the study through newsletter mailings, website postings, and social media posts. Recruitment was conducted via the aforementioned methods from October 2023 to June 2024. Eligibility criteria, including age, level of schooling, and diagnosis of KS, were confirmed via self‐report from the participant or their guardian prior to scheduling the interview. Informed consent was obtained from all participants over the age of 18 years. Informed assent and parent/guardian consent were obtained for all participants under age 18 years. Participants received a $50 Visa gift card upon completion of all study procedures.

### 2.2 Procedures and instrumentation

Data from this study were collected via semi‐structured interviews assessing the following three areas: (1) resources and support systems AYA with KS utilize to cope with challenges related to their condition, (2) psychosocial well‐being of AYA with KS and how KS is perceived to impact it, and (3) attitudes of AYA with KS about testicular dysfunction, specifically the likely need for testosterone replacement and fertility‐related interventions. The current study reports data on the first and second areas.

The authors collaboratively developed an interview guide (Data S1) to assess these content areas based on three primary factors: (1) an interview guide developed by Papadakis et al. (2021) exploring fertility‐related healthcare and decision‐making needs for AYA with DSDs, (2) literature review (Dennis et al., 2015; Richardson et al., 2022; Turriff et al., 2015, 2017), and (3) the authors' expertise caring for children and AYA with KS in a multidisciplinary clinic at a single pediatric institution (AW—genetic counselor, CF—endocrinologist, and JP—pediatric psychologist). Individuals with KS make up a significant portion of the patient population that authors AW, CF, and JP care for, which contributed to their motivation to explore the topics in this study to contribute to the development of comprehensive care for this patient population. Additionally, the research team included individuals with qualitative research expertise along with experience in genetic counseling.

Semi‐structured interviews were completed by one author (AT) with each consented participant between November 2023 and June 2024. At the time of the interviews, female interviewer, AT, was a graduate student at a genetic counseling program. The interviewer did not have a treatment relationship with any participants. All interviews were conducted online via Zoom (version 5.16.10). Participants were encouraged to conduct the interview in a private space with the option to turn their video on or off for the interview, while the interviewer's video remained on. All interviews were audio recorded, assigned a study ID, and subsequently transcribed by one author (AT) with identifying information, such as first names, places of employment, and names of schools removed. Demographic information, including sex and race/ethnicity, was collected via self‐report by the participant during the interview.

### 2.3 Data analysis

Transcripts were analyzed by authors AT, JP, and AW using an inductive and latent approach with qualitative data analysis software, Dedoose Version 9.0.107 (2023), with AT as the primary coder. AT had taken coursework in qualitative data analysis, and JP and AW have experience in qualitative data analysis. The data were approached from a social constructivist lens, which acknowledges that personal experiences create multiple realities (Cresswell & Poth, 2018). Data were analyzed using reflexive thematic analysis following the six steps outlined by Braun and Clarke (2006, 2020, 2022) (Pope et al., 2020). The reflexive thematic analysis approach was utilized because it took into account the positionality of the researchers and helped capture an authentic representation of the participants' experiences. The researchers first familiarized themselves with the data by reviewing the transcripts. The second step of generating initial codes was conducted independently using an inductive approach. During this time, AT, JP, and AW met regularly to discuss preliminary descriptive codes and determine what ideas needed further exploration. Data collection was concluded after 13 interviews as sufficient informational power was reached. This was determined by the researchers based on the specificity of the participant population, in depth interview data including strong quality of dialogue, and targeted aims of the study (Malterud et al., 2015; Wainstein et al., 2022). Discussion then continued between AT, JP, and AW to refine the preliminary codes by collapsing those that shared common meanings into one code and were able to generate initial themes (step 3). The fourth and fifth steps involved reviewing the themes through discussion in several meetings. The final themes were reported as step six after themes were clarified by the research team. Quotes were minimally edited for readability only.

## 3 RESULTS

### 3.1 Demographics

Thirteen male‐identified individuals with KS participated. Participant demographics and interview details are described in Table 1.

**TABLE 1:** Participant demographic and interview characteristics.

Characteristic	*N*	%
Sex		
Male	13	100
Age at interview (years)		
14–17	7	54
18–23	6	46
Age at Interview, years median (range)		16 (14–23)
Race/ethnicity		
White	12	92
Asian (Pakistani)	1	8
Level of education		
In High School	7	54
In College	5	38
Completed College	1	8
Recruitment source		
Support group	6	46
Pediatric Institution	7	54

Length of interview	
Age at interview (years)	Median length, min (range)
14–17	27 (17–41)
18–23	38 (27–44)
All Interviews	31 (17–44)

### 3.2 Themes

Three themes were generated regarding psychosocial impacts of KS and support systems AYA with KS utilize to cope with challenges of their diagnosis: (1) “I'm a little different”—rejecting stigmatization and integrating a diagnosis of KS into one's identity, (2) “Let it out”—lessons learned when disclosing one's diagnosis of KS to others, and (3) “They accept me for who I am”—the building blocks of a support system.

#### 3.2.1 “I'm a little different”—rejecting stigmatization and integrating a diagnosis of KS into one's identity

A feeling of stigmatization associated with having an extra chromosome was commonly described by participants. Specifically, participants shared their perception that others view KS as a disability and personally rejected such a label, stating “I just wish that more people would not look at Klinefelters as ‘disabled.’ I feel like when you go to a doctor and they're like, ‘you have Klinefelters, you have an extra chromosome’ people just automatically assume disabled, but that's not the case” (ID#5, 21 years). This feeling of stigmatization was described by many participants as something that was more prominent when they first learned about their diagnosis and has decreased over time. This is accompanied by participants growing more comfortable with integrating their diagnosis into their identity over time, saying “at the time I thought there was something wrong with me. At the end of the day it's a difference, yes, but it's not a major one I guess, in my experience at least” (ID#4, 20 years) and “I used to be really scared about it. I was like ‘I don't want people knowing I have a disability.’ But now, after high school, I haven't really cared too much about keeping it a secret.” (ID#13, 21 years).

Many participants stated they do not feel KS plays a large role in how they view themselves, stating “I'm a little different from other people” (ID#6, 16 years), it is “just a part of my life” (ID#13, 21 years), and “it's a fun little quirk” (ID#4, 20 years). There was also an emphasis that people would not be able to tell that a participant has KS by physical appearance alone, stating “looking from the outside, there's not much of a difference” (ID#4, 20 years). Overall, participants largely viewed KS as “a part of who I am” (ID#12, 16 years) and not representative of their entire self.

#### 3.2.2 “Let it out”—lessons learned when disclosing one's diagnosis of KS to others

All 13 participants had disclosed their KS diagnosis to at least one individual, however, what specifically was shared and how much detail was provided varied. Some participants do not use the term “Klinefelter Syndrome” when disclosing and instead focus on medical aspects of their diagnosis such as testosterone treatment or infertility. For example, regarding disclosure to friends, one participant stated, “I don't know if this sounds kind of weird but when bodybuilders say they're taking testosterone I say, ‘Haha I take this’,” but when asked if he had told any friends that he has KS, this participant stated “no, not specifically Klinefelter” (ID#7, 21). There was also variability in whether participants were more likely to have shared broad information about KS, for example, one participant stated “my therapist said just describe it as a rare, I guess, chromosome condition” (ID#3, 15 years) versus more detailed information regarding symptoms they attribute to KS or anticipate experiencing as a result of the condition with most participants having chosen the latter. For example, when one participant was asked what he recalls telling his friends about KS, he stated, “just like the rundown […] just basic things that come with it. Like I take testosterone for it, without it I'm really tired […] and just my English is not that good because of it. Just things along those lines” (ID#13, 21 years) and another stating “I pretty much told [my girlfriend] everything that the doctor said […] like what goes on with it and how it works, what could happen, what could be the side effects, if I'm being on this what could happen with that. That type of stuff” (ID#6, 16 years). Concerning reactions when disclosing, the majority of participants described feeling “a sense of relief” (ID#1, 23 years) after disclosing to others despite feeling that “at first, [disclosing] was a little bit scary” (ID#13, 21 years), with one participant describing his disclosure experience as “kind of freeing in a way – knowing [my friends] know who I am” (ID#11, 16 years) and another stating “you know how you get really nervous if you're confessing something, or it's like your heart is racing but once you say it you're more at ease” (ID#2, 21 years).

**FIGURE 1:**
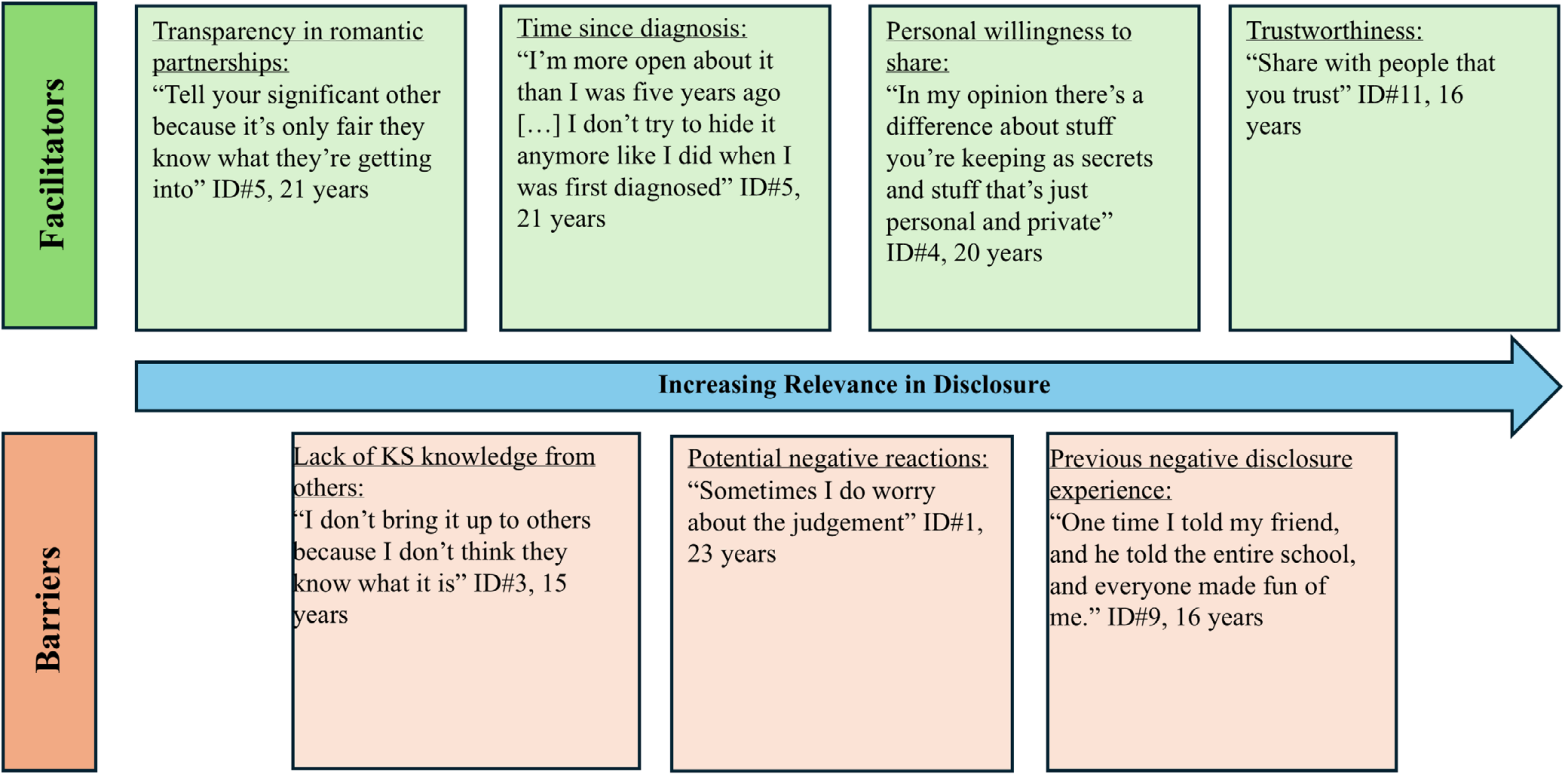
Perceived facilitators and barriers to disclosing Klinefelter syndrome (KS) diagnosis to others in order of increasing relevance. Level of relevance was based on the consistency with which this factor was mentioned across participants.

A summary of the facilitators and barriers to disclosure reported by participants can be seen below (Figure 1). The facilitators and barriers are listed in order of increasing relevance based on how frequently a given facilitator or barrier was mentioned by participants.

The facilitator that was identified by all participants in disclosing one's KS diagnosis was how trustworthy the participant perceived the individual. Many specified they would disclose to “just best friends […] people I can trust” (ID#8, 14 years). Individual personality was also noted to have played a role in the decision to disclose with participants acknowledging “it depends kind of on personality, I'm a very easy going, joking person” (ID#4, 20 years) or “I'm not really one to share too many personal details […] I'm just a private person” (ID#1, 23 years). Additionally, the amount of time since their initial diagnosis of KS played a role in disclosure, with participants having noted an increased comfort level disclosing their diagnosis of KS to others as time went on, with one participant stating, “at first sharing was a little weird and awkward, back then I would really only share with my group of close friends, then I just kind of joked about it” (ID#4, 20 years). For fertility‐related information, participants described wanting to be “up front” with romantic partners (ID#5, 21 years) and were more likely to discuss fertility in the context of seeking emotional support (ID#1, 23 years; ID#2, 21 years).

The greatest barrier to disclosing one's diagnosis was worries about potential reactions from others. Participants cited worries about anticipated “rumors starting” (ID#6, 16 years), “judgment” (ID#1, 23 years), or thoughts that “kids might pick on you” (ID#10, 16 years) as factors that prevented them from disclosing. Additionally, previous negative reactions from others discouraged participants from wanting to disclose in the future, for example, “the friend I had in high school cried and it's something that sticks with you. So it's hard to tell other people because you think that's gonna be the reaction and you just don't want that” (ID#7, 21 years). Another participant detailed a negative disclosure experience that deterred him from wanting to disclose to others stating, “it's an extra chromosome right, and a lot of people like to make jokes about 47 chromosomes, you know, derogatory jokes so that makes me not want to talk about it. That's why I just keep it within my family” (ID#12, 16 years). One participant also worried that others might try to put a “positive spin on things” such as “someone trying to cheer me up but in a way that's putting me down. Like someone saying ‘you don't need kids’ or ‘that's a good thing’ but since I want kids that would be a negative thing” (ID#2, 21 years). Another barrier to disclosure was that participants felt like others would not know what KS is. To overcome this barrier, participants advised taking the explanation step by step when discussing the condition to someone and to personally do additional research on KS to better educate others. As one participant emphasized, disclosing KS to others is “all a learning experience” (ID#8, 14 years).

#### 3.2.3 “They accept me for who I am”—The building blocks of a support system

**FIGURE 2:**
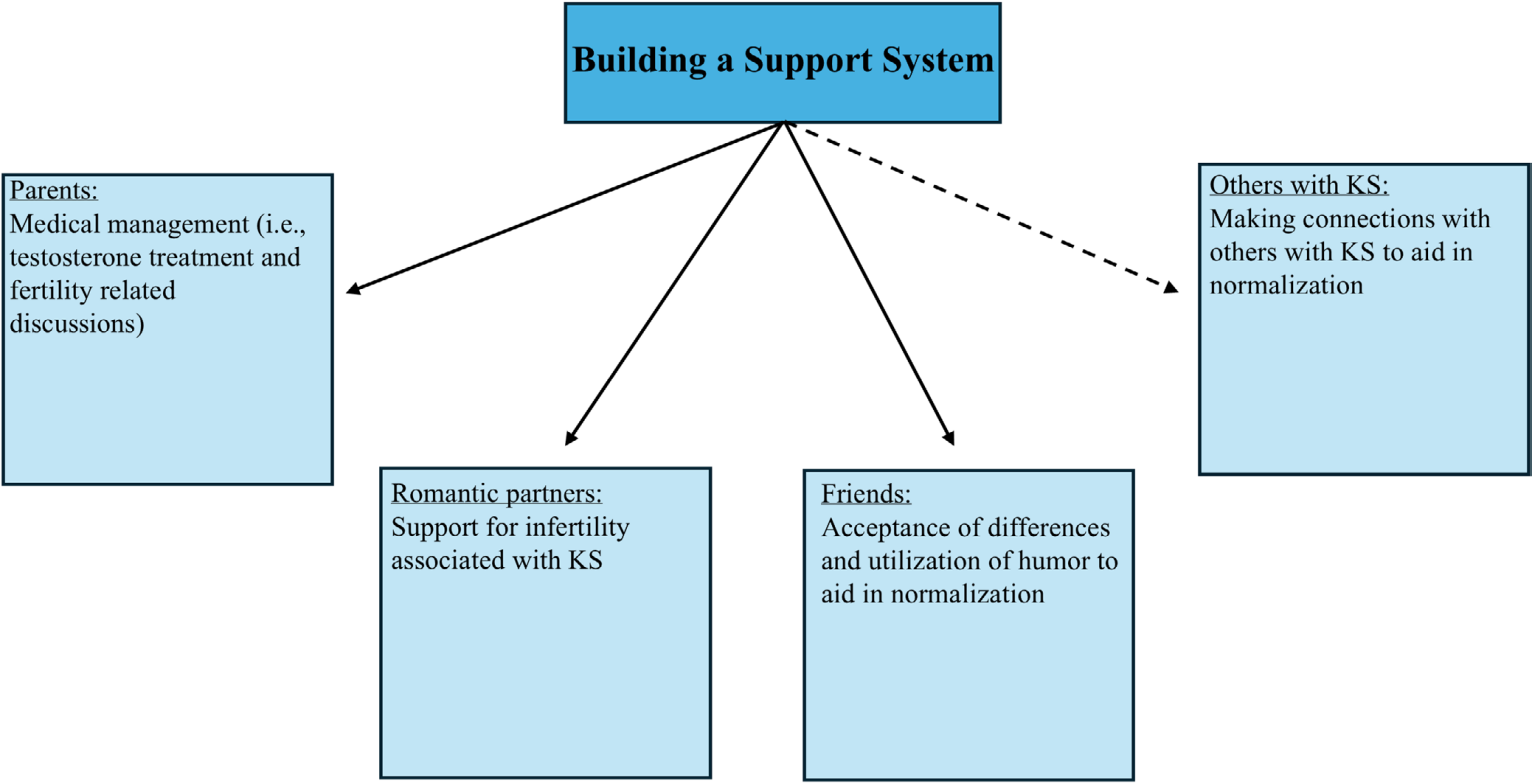
Components of participants' support systems and how these relationships are utilized for various aspects of support. Solid lines represent established relationships with support people. Dashed lines represent potential sources of support. Klinefelter syndrome (KS).

Participants identified several relationships that they utilized as sources of support for various aspects of KS (Figure 2). Participants named a mix of one or both parents (*n* = 10), romantic partners (*n* = 4), and friends (*n* = 8) as important members of their support system.

Parents primarily served as a support to participants regarding medical issues related to KS, specifically gynecomastia, infertility, and testosterone treatments. Participants reported various displays of support, whether that be attending doctor's visits (ID#12, 16 years), researching information about the condition (ID#13, 21 years), or facilitating treatment decisions: “[my mom] left the decisions [whether or not to start testosterone] up to me but highly suggested it, which I'm thankful for” (ID#6, 16 years). Most participants felt comfortable talking to their parents about the medical aspects of KS, however, one noted that it was “really hard in the beginning to talk to my mother about how my testicles are small” (ID#5, 21 years). There was not a distinct difference in the number of participants who sought support from their mother, father, or both parents.

Romantic partners were identified as important members of support systems for all participants who reported they were currently in a romantic relationship (*n* = 4; all identified their partner as a “girlfriend”). Participants largely felt that their disclosure experiences to their girlfriends were positive and nonjudgmental with the focus being on information about infertility. Participants, regardless of if they currently had a partner or not, felt romantic relationships would be the most impacted by KS due to infertility stating, “that's the only thing really that I think would come up” (ID#4, 20 years). However, impacts to romantic relationships were more anticipatory as none of the participants were currently having family planning discussion with their girlfriends with one participant expressing, “it hasn't [impacted the relationship] yet because I'm still young and not thinking about children at the moment” (ID#7, 19 years). One participant voiced that they feel “a bit more apathetic towards relationships” (ID#11, 16 years) due to infertility.

Friends were also mentioned by most participants (*n* = 8) as part of their support system, primarily for normalization, acceptance of their differences, and for some, the utilization of humor. Participants describe minimal reactions from friends, stating “they accept me for who I am and don't let it get in the way” (ID#1, 23 years) and “I'm just a regular guy, so it hasn't really impacted [friendships] at all” (ID#13, 21 years). Many participants reported that they experienced relief in finding humor about KS with friends, “I just joke with them about it. It helps me think of it as something not so serious” (ID#7, 19 years). Overall, there was a strong emphasis that participants wanted to feel like they fit in with friends, specifically that they would not have differences held against them and they could “be myself” (ID#8, 14 years). While many participants appreciated finding humor about KS with their friends, others expressed that such humor could sometimes be received as mean‐spirited, with youths feeling stigmatized saying, “I made it very clear like I'm not gonna be friends with you if you make jokes about [KS]” (ID#12, 16 years).

Engagement with the greater KS community, specifically other individuals with KS and formal support/community organizations for KS, varied among participants. Eight participants were not engaged in support/community organizations related to KS and mostly cited “there's no severe problems or anything” (ID#2, 21 years) that would have prompted them to seek support. Participants also stated that joining a community organization is something “I just haven't really thought about” (ID#13, 21 years) or that there may have been issues with “time management” (ID#12, 16 years). Two of the participants who were connected to support groups via Facebook describe passive participation stating they “haven't really talked in that group much” and instead “just like to read and see what other people are doing” (ID#1, 23 years). Some participants recruited through Living With XXY did describe activities that show they were active in the KS community such as writing poems to share with their class (ID#6, 14 years), creating podcasts (ID#9, 16 years), giving interviews (ID#11, 16 years), or participating in research (ID#11, 16 years; ID#12, 16 years). Half of the participants recruited through Living With XXY did not describe themselves as being members of a support group, instead identifying their mothers as active members. Parents helped facilitate participant's engagement in community organizations, with one participant stating, “I'm part of a support group only because my mom is a part of it” (ID#12, 16 years).

Most participants said they do not feel a sense of community with others with KS, with the main reason being not knowing anyone else with KS, with one participant stating, “I don't know anyone in my real life who also has it but I feel like if I knew it'd definitely be a sense of community” (ID#12, 16 years). However, participants voiced that social media is a way to help foster connection, “I still haven't met anyone that has it so never felt that connection. But I see on social media that people have it, people are doing good things about it so I feel some connection there” (ID#13, 21 years).

Despite many participants not having felt a sense of community with others with KS, participants overall felt that receiving a diagnosis of KS was validating, such as, “putting the name Klinefelter syndrome with me, it just gave an answer to a lot of things that I had [physical appearance, ADD, and Tourette's]” (ID#7, 21 years). Additionally, participants found a sense of normalization in knowing “that I'm not the only person that has it. A lot of people might have it but don't know they have it” (ID#2, 21 years).

## 4 DISCUSSION

### 4.1 Summary

The current study used qualitative methods to explore the psychosocial well‐being of AYA with KS. AYA with KS shared how they integrate their diagnosis into their identity, experiences with disclosing their diagnosis to others, and insights into their support system. We report several themes that can inform current clinical management, specifically regarding counseling AYA with KS in fostering a sense of positive self‐concept and building an effective support system.

### 4.2 Comparison with literature and practice implications

Participants in this study described feelings of stigmatization associated with having an extra chromosome and largely rejected both this stigma and the label of KS as a disability by others. This is consistent with previous research, which found that individuals of all ages with KS do not perceive themselves to be stigmatized as a result of the condition and that perceived stigma was not found to be correlated with adaptation, or coming to terms with the implications of KS (Bender et al., 2001; Bourke et al., 2014; Turriff et al., 2015). Additionally, in previous research involving AYA with DSDs, individuals found that psychological care helped them make meaning of their variation (Callens et al., 2021; Lundberg et al., 2016). Being able to integrate a diagnosis of KS into one's identity is important in helping AYA with KS make meaning of their diagnosis. This in turn can allow individuals to create a narrative regarding their condition over time.

Participants in the present study found that the ability to integrate KS into their identity and make meaning of one's diagnosis was easier or occurred naturally over time, with many feeling a greater sense of stigma upon first learning about their diagnosis. However, earlier research among those with X/Y chromosome aneuploidy has shown that patients who were able to access up‐to‐date information and supportive resources were better able to emotionally process and develop resiliency following their diagnosis (Gallo et al., 2010; Richardson et al., 2022). Additionally, previous research in patients with DSDs found that when patients lack knowledge about their condition it affects both the frequency and level of depth with which they talk about it, with patients expressing that they wish they would have been taught everyday language to use to talk about their DSD (Gallo et al., 2010; Media et al., 2022). Studies have shown that medical providers, including genetic counselors, can facilitate this type of education and leave patients with a better understanding of their condition (Engberg et al., 2016; Jaramillo et al., 2019; Riggan et al., 2020, 2021; Sanders & Carter, 2015; van Engelen et al., 2011).

Ensuring that AYA with KS have a clear understanding of their diagnosis and how it contributes to their own identity is crucial as this has been shown to play a role in whether or not an individual will share their KS with others (Herlihy, Halliday, et al., 2011; Herlihy, McLachlan, et al., 2011). The present study indicates that sharing one's diagnosis with trusted people has an overall positive impact on the participant. Earlier studies have shown that barriers to disclosing one's DSD with others include fears about being perceived as different, which affected the balance between concealing and revealing personal information (Herlihy, Halliday, et al., 2011; Herlihy, McLachlan, et al., 2011). Participants in this study had similar worries about the potential judgment and stigma they may face by sharing details about having KS with others; however, these worries seemed to diminish over time as participants were better able to integrate these differences into their sense of self. It is important to acknowledge that the personality of the individual patient plays a role in the information they share about their diagnosis with others. Emphasizing that it is okay for a patient to share as much or as little about KS as they would like is important. Additionally, equipping AYA with KS with the language needed to feel confident in disclosing to others, if desired, is essential.

While the actual content of what was disclosed varied, the act of disclosing one's diagnosis of KS to others in their trusted support system generally provided relief. This is consistent with earlier research, which has shown that the versions of disclosure, specifically the amount of details shared, can vary depending on the closeness of the person being disclosed to (Callens et al., 2021). The present study found that one factor that may affect what information is shared with an individual is the role that individual plays in the participant's support system. Specifically, participants in this study identified romantic partners as being the most likely to be affected by KS due to the associated infertility and consequently disclosed their diagnosis to their romantic partners, thereby integrating them into their support system for coping with challenges related to KS. While prior research has shown that AYA with DSDs felt like their infertility had a significant impact on their relationships, with some even feeling as though they had already disappointed their future partners (Callens et al., 2021), those feelings were not shared by AYA with KS in the present study. Rather, participants in this study largely did not feel as though it has a significant impact on their current romantic partners, viewing it instead as a potential future scenario perhaps due to participants' younger ages. In addition to romantic partners, many participants in the present study indicated that their parents play an important role in their support system, with their main role being assisting with medical aspects of KS. As is demonstrated in this study as well as in previous research, it is important for patients to have a diverse network of support people including romantic partners, parents, and friends as those with higher levels of social support have less psychological distress (Bender et al., 1995; Lundberg et al., 2016).

Previous research has shown that an important part of the support system for adults (mean age 45.3 years) with various X and Y chromosome aneuploidies is others who have been previously diagnosed with the same condition. These individuals are viewed as a valuable source of support and information about potential challenges associated with their condition (Richardson et al., 2022). However, it is important to note that participants in the aforementioned study were recruited through a support organization (Association for X and Y Chromosome Variations). In the present study, participants largely did not identify others with KS as part of their support system, and most did not actively engage in support groups. Rather, participants were more likely to identify passive engagement with others with KS on social media as helpful to them in feeling a positive connection. Even those participants recruited through a support organization were more likely to identify their parents as members of the group rather than themselves. This suggests that simply providing patients a list of support groups is not sufficient to foster active engagement among AYA with KS. Additionally, several participants in this study mentioned that they do not feel like they have serious issues related to KS that would prompt them to seek support. This may suggest a misunderstanding of what to expect from support groups. Therefore, it could be beneficial for providers to share information about support groups in a way that highlights the diversity of experiences, benefits, and resources that one may expect from participation with such an organization, with an emphasis on providing opportunities to connect with others with KS. Overall, there is room for improvement in connecting these patients with peers (Callens et al., 2021).

The findings in the present study along with their suggested clinical considerations are summarized in Table 2. Additional research is needed to assess if the study findings can be replicated in a larger cohort and best determine appropriate practice implications.

**TABLE 2:** Clinical considerations for providers caring for AYA patients with KS.

Study finding	Clinical consideration
Theme 1: “I'm a little different”—Rejecting stigmatization and integrating a diagnosis of KS into one's identity	Assist patients with adaptively integrating KS into their sense of identity and self‐esteem while equipping patients with tools to decrease feelings of stigma surrounding their diagnosis
Theme 2: “Let it out”—Lessons learned when disclosing one's diagnosis of KS to others	Emphasize that it is okay for a patient to share as much or as little about their diagnosis as they feel comfortable with and to focus on sharing with people they trust
	Prepare patients for disclosure to others by ensuring they feel confident in both their knowledge of KS and how to explain KS to others
Theme 3: “They accept me for who I am”—The building blocks of a support system	Help patients evaluate if their support system is meeting their needs and offer recommendations for strengthening it, for example, by including people like parents, romantic partners, and friends
	Search for ways to help patients actively engage in support networks and assist patients in identifying how connecting with the KS community may benefit them. Simply providing a list of support organizations may not be sufficient

### 4.3 Limitations

Potential biases were introduced through recruiting participants from a single academic pediatric medical center located in an urban area. Additionally, recruitment via a support group may bias results toward positive views of support groups for participants recruited through that organization. This study also had a lack racial and ethnic diversity as most participants identified as white. The perspectives of people of color with KS may have different views that were not accounted for by participants in the current study and further research including more diverse samples is needed.

## 5 CONCLUSIONS

This study qualitatively explored AYA perspectives on the psychosocial impact of KS and the support systems AYA with KS utilize to cope with the challenges of their diagnosis. Results revealed potential areas for improvement, such as assisting AYA with KS in integrating their diagnosis into their identity and decreasing potential feelings of stigma associated with KS. This would contribute to facilitating disclosure to others and in turn assist AYA with KS in building a varied support system. Additionally, this study identified a need to understand how to actively engage AYA with KS in support groups and connect them to others with KS. Informed and comprehensive pediatric care for individuals with KS is more important than ever given the increasing young population of patients diagnosed with KS. Results of this study aim to help provide a foundation for future research and educational and clinical interventions to better support this growing patient population in adolescence and young adulthood.

## AUTHOR CONTRIBUTIONS

**Abigail Tubman:** conceptualization, data curation, formal analysis, funding acquisition, investigation, methodology, project administration, visualization, writing – original draft preparation, writing – review and editing. **Jaclyn L. Papadakis:** conceptualization, methodology, formal analysis, resources, data curation, writing – review and editing, visualization, supervision, project administration. **Courtney Finlayson:** conceptualization, methodology, resources, writing – review and editing, visualization, supervision, project administration, funding acquisition. **Debra Duquette:** conceptualization, methodology, resources, writing – review and editing, visualization, supervision, project administration. **Allison Goetsch Weisman:** conceptualization, methodology, formal analysis, resources, data curation, writing – review and editing, visualization, supervision, project administration.

## CONFLICT OF INTEREST STATEMENT

All authors declare that they have no conflicts of interest.

## ETHICS STATEMENT

Human Studies and Informed Consent: This research was reviewed by the Ann & Robert H. Lurie Children's Hospital of Chicago's Institutional Review Board and was granted approval in September 2023 (2023‐6234). All procedures followed were in accordance with the ethical standards of the responsible committee on human experimentation (institutional and national) and with the Helsinki Declaration of 1975, as revised in 2000. Written informed consent was obtained from all participants over 18 years of age prior to their inclusion in this study. Written informed assent was obtained from all participants under 18 years of age, and written informed consent was obtained from their parent/guardian prior to their inclusion in this study.

Animal Studies: No nonhuman animal studies were carried out by the authors for this article.

## Supporting information


Data S1:

Data S2:

## Data Availability

Research data are not shared.

## References

[jgc470074-bib-0001] Abramsky, L. , & Chapple, J. (1997). 47,XXY (Klinefelter syndrome) and 47,XYY: Estimated rates of and indication for postnatal diagnosis with implications for prenatal counselling. Prenatal Diagnosis, 17, 363–368. 10.1002/(SICI)1097-0223(199704)17:4<363::AID-PD79>3.0.CO;2-O 9160389

[jgc470074-bib-0002] Aksglaede, L. , Davis, S. M. , Ross, J. L. , & Juul, A. (2020). Minipuberty in Klinefelter syndrome: Current status and future directions. American Journal of Medical Genetics, 184, 320–326. 10.1002/ajmg.c.31794 32476267 PMC7413638

[jgc470074-bib-0003] Bearelly, P. , & Oates, R. (2019). Recent advances in managing and understanding Klinefelter syndrome. F1000Research, 8, 112. 10.12688/f1000research.16747.1 PMC6352920 30755791

[jgc470074-bib-0004] Bender, B. , Harmon, R. , Linden, M. , & Robinson, A. (1995). Psychosocial adaptation of 39 adolescents with sex chromosome abnormalities. Pediatrics, 96, 302–308. 10.1542/peds.96.2.302 7630689

[jgc470074-bib-0005] Bender, B. , Linden, M. , & Harmon, R. (2001). Life adaptation in 35 adults with sex chromosome abnormalities. Genetics in Medicine, 3, 187–191. 10.1097/00125817-200105000-00007 11388759

[jgc470074-bib-0006] Berglund, A. , Viuff, M. H. , Skakkebaek, A. , Chang, S. , Stockholm, K. , & Gravholt, C. H. (2019). Changes in the cohort composition of turner syndrome and severe non‐diagnosis of Klinefelter, 47,XXX and 47,XYY syndrome: A nationwide cohort study. Orphanet Journal of Rare Diseases, 14, 16. 10.1186/s13023-018-0976-2 30642344 PMC6332849

[jgc470074-bib-0007] Bojensen, A. , Juul, S. , & Gravholt, C. H. (2003). Prenatal and postnatal prevalence of Klinefelter syndrome: A National Registry Study. The Journal of Clinical Endocrinology and Metabolism, 88, 622–626. 10.1210/jc.2002-021491 12574191

[jgc470074-bib-0008] Bourke, E. , Snow, P. , Herlihy, A. , Amor, D. , & Metcalfe, S. (2014). A qualitative exploration of mothers' and fathers' experiences of having a child with Klinefelter syndrome and the process of reaching this diagnosis. European Journal of Human Genetics, 22, 18–24. 10.1038/ejhg.2013.102 23695282 PMC3865389

[jgc470074-bib-0009] Braun, V. , & Clarke, V. (2006). Using thematic analysis in psychology. Qualitative Research in Psychology, 3, 77–101. 10.1191/1478088706qp063oa

[jgc470074-bib-0010] Braun, V. , & Clarke, V. (2020). Can I use TA? Should I use TA? Should I not use TA? Comparing reflexive thematic analysis and other pattern‐based qualitative analytic approaches. Counselling and Psychotherapy Research, 21(1), 37–47. 10.1002/capr.12360

[jgc470074-bib-0011] Braun, V. , & Clarke, V. (2022). Is thematic analysis used well in health psychology? A critical review of published research, with recommendations for quality practice and reporting. Health Psychology Review, 17(4), 695–719. 10.1080/17437199.2022.2161594 36656762

[jgc470074-bib-0012] Callens, N. , Kreukels, B. , & van de Grift, T. (2021). Young voices: Sexual health and transition care needs in adolescents with Interesx/differences of sex development. Journal of Pediatric and Adolescent Gynecology, 34, 176–189. 10.1016/j.jpag.2020.11.001 33181339

[jgc470074-bib-0013] Chang, S. , Skakkebaek, A. , Davis, S. , & Gravholt, C. H. (2020). Morbidity in Klinefelter syndrome and the effect of testosterone treatment. American Journal of Medical Genetics, 184, 344–355. 10.1002/ajmg.c.31798 32496001 PMC7413637

[jgc470074-bib-0014] Cresswell, J. W. , & Poth, C. N. (2018). Qualitative inquiry and research design: Choosing among five approaches (4th ed.). SAGE.

[jgc470074-bib-0015] Dennis, A. , Howell, S. , Cordeiro, L. , & Tartaglia, N. (2015). “How should I tell my child?” disclosing the diagnosis of sex chromosome aneuploidies. Journal of Genetic Counseling, 24, 88–103. 10.1007/s10897-014-9741-4 25179748 PMC5340499

[jgc470074-bib-0016] Engberg, H. , Moller, A. , Hagenfeldt, K. , Nordenskjold, A. , & Frisen, L. (2016). The experience of women living with congenital adrenal hyperplasia: Impact of the condition and the care given. Clinical Endocrinology, 85, 21–28. 10.1111/cen.13054 26941069

[jgc470074-bib-0018] Fjermestad, K. W. , Huster, R. , Thunberg, C. , Stokke, S. , Gravholt, C. H. , & Solbakk, A. (2020). Neuropsychological functions, sleep, and mental health in adults with Klinefelter syndrome. American Journal of Medical Genetics, 184, 482–492. 10.1002/ajmg.c.31797 32415904

[jgc470074-bib-0019] Gallo, A. , Knafl, K. , & Angst, D. (2010). Information Management in Families who have a child with a genetic condition. Journal of Pediatric Nursing, 24, 194–204. 10.1016/j.pedn.2008.07.010 PMC2735554 19467432

[jgc470074-bib-0020] Herlihy, A. , Halliday, J. , Cock, M. , & McLachlan, R. (2011). The prevalence and diagnosis rates of Klinefelter syndrome: An Australian comparison. The Medical Journal of Australia, 194, 24–28. 10.5694/j.1326-5377.2011.tb04141.x 21449864

[jgc470074-bib-0021] Herlihy, A. , McLachlan, R. , Gillam, L. , Cock, M. , Collins, V. , & Halliday, J. (2011). The psychosocial impact of Klinefelter syndrome and factors influencing quality of life. Genetics in Medicine, 13, 632–642. 10.1097/GIM.0b013e3182136d19 21546843

[jgc470074-bib-0022] Hughes, I. A. , Hours, C. , Ahmed, S. F. , & Lee, P. A. (2006). Consensus statement on management of intersex disorders. Journal of Pediatric Urology, 2, 148–162. 10.1016/j.jpurol.2006.03.004 18947601

[jgc470074-bib-0023] Janusz, J. , Harrison, C. , Boada, C. , Cordeiro, L. , Howell, S. , Tartaglia, N. , & Boada, R. (2020). Executive function in XXY: Comparison of performance‐based measures and rating scales. American Journal of Medical Genetics, 184, 469–481. 10.1002/ajmg.c31804 32519473 PMC8363474

[jgc470074-bib-0024] Jaramillo, C. , Nyquist, C. , Riggan, K. , Egginton, J. , Phelan, S. , & Allyse, M. (2019). Delivering the diagnosis of sex chromosome aneuploidy: Experiences and preferences of parents and individuals. Clinical Pediatrics, 58, 336–342. 10.1177/0009922818817310 30516062

[jgc470074-bib-0025] Lundberg, T. , Roen, K. , Hirschberg, A. , & Frisen, L. (2016). “It's part of me, not all of me”: Young Women's experiences of receiving a diagnosis related to diverse sex development. Journal of Pediatric and Adolescent Gynecology, 29, 338–343. 10.1016/j.jpag.2015.11.009 26639995

[jgc470074-bib-0026] Malterud, K. , Siersma, V. D. , & Guassora, A. D. (2015). Sample size in qualitative interview studies: Guided by information power. Qualitative Health Research, 26(13), 1753–1760. 10.1177/1049732315617444 26613970

[jgc470074-bib-0028] Media, L. M. , Fauske, L. , Sigurdardottir, S. , Feragen, K. J. B. , Heggeli, C. , & Waehre, A. (2022). ‘It was supposed to be a secret’: A study of disclosure and stigma as experienced by adults with differences of sex development. Health Psychology and Behavioral Medicine, 10, 579–595. 10.1080/21642850.2022.2102018 35898596 PMC9310795

[jgc470074-bib-0029] Papadakis, J. , Poquiz, J. , Buchanan, C. , Chan, Y.‐M. , Crerand, C. , Hansen‐Moore, J. , Kapa, H. , Nahata, L. , & Pratt, K. (2021). Fertility discussions: Perspectives of adolescents and young adults with differences of sex development. Clinical Practice in Pediatric Psychology, 9, 372–383. 10.1037/cpp0000373 35310824 PMC8932642

[jgc470074-bib-0030] Pope, C. , Ziebland, S. , & Mays, N. (2020). Analysis. In C. Pope & N. Mays (Eds.), Qualitative research in health (4th ed., pp. 111–134). John Wiley & Sons Ltd.

[jgc470074-bib-0031] Richardson, J. , Ahlawat, N. , Riggan, K. , Close, S. , & Allyse, M. (2022). Experiences of individuals receiving a sex chromosome multisomy diagnosis. Journal of Community Genetics, 13, 619–628. 10.1007/s12687-022-00604-0 35986191 PMC9681968

[jgc470074-bib-0032] Riggan, K. , Close, S. , & Allyse, M. (2020). Family experiences and attitudes about receiving the diagnosis of sex chromosome aneuploidy in a child. American Journal of Medical Genetics, 184, 404–413. 10.1002/ajmg.c.31781 32181570 PMC7321881

[jgc470074-bib-0033] Riggan, K. , Gross, B. , Close, S. , Weinberg, A. , & Allyse, M. (2021). Prenatal genetic diagnosis of a sex chromosome aneuploidy: Parent experiences. Journal of Genetic Counseling, 30, 1407–1417. 10.1002/jgc4.1407 33723878

[jgc470074-bib-0034] Sanders, C. , & Carter, B. (2015). A qualitative study of communication between young women with disorders of sex development and health professionals. Journal of Advanced Nursing, 2015, 1–7. 10.1155/2015/653624 PMC4682458 25893820

[jgc470074-bib-0035] Skakkebaek, A. , Moore, P. J. , Pedersen, A. D. , Bojessen, A. , Kristensen, K. , Fedder, J. , Hertz, J. M. , Ostergaard, J. R. , Wallentin, M. , & Gravholt, C. H. (2018). Anxiety and depression in Klinefelter syndrome: The impact of personality and social engagement. PLoS One, 9, e0206932. 10.1371/journal.pone.0206932 PMC6226182 30412595

[jgc470074-bib-0036] Turriff, A. , Levy, H. , & Biesecker, B. (2011). Prevalence and psychosocial correlates of depressive symptoms among adolescents and adults with Klinefelter syndrome. Genetics in Medicine, 13, 966–972. 10.1097/GIM.0b013e3182227576 21799429 PMC3208082

[jgc470074-bib-0037] Turriff, A. , Levy, H. , & Biesecker, B. (2015). Factors associated with adaptation to Klinefelter syndrome: The experience of adolescents and adults. Patient Education and Counseling, 98, 90–95. 10.1016/j.pec.2014.08.012 25239793 PMC5160995

[jgc470074-bib-0038] Turriff, A. , Mcnamara, E. , Levy, H. , & Biesecker, B. (2017). The impact of living with Klinefelter syndrome: A qualitative exploration of adolescents and adults. Journal of Genetic Counseling, 26, 728–737. 10.1007/s10897-016-0041-z 27832510 PMC5425317

[jgc470074-bib-0039] van Engelen, K. , Baars, M. , van Rongen, L. , van der Velde, E. , Mulder, B. , & Smets, E. (2011). Adults with congenital heart disease: Patients' knowledge and concerns about inheritance. American Journal of Medical Genetics, 155, 1661–1667. 10.1002/ajmg.a.34068 21671389

[jgc470074-bib-0040] Wainstein, T. , Elliot, A. M. , & Austin, J. C. (2022). Considerations for the use of qualitative methodologies in genetic counseling research. Journal of Genetic Counseling, 32(2), 300–314. 10.1002/jgc4.1644 36271905

[jgc470074-bib-0041] Zganjar, A. , Nangia, A. , Sokol, R. , Ryabets, A. , & Samplaski, M. (2020). Fertility in adolescents with Klinefelter syndrome; a survey of current clinical practice. Journal of Clinical Endocrinology and Metabolism, 105, dgz044. 10.1210/clinem/dgz044 31608942

[jgc470074-bib-0042] Zitzmann, M. , Aksglaede, L. , Corona, G. , Isidori, A. , Juul, A. , T'Sjoen, G. , Kliesch, S. , D'Hauwers, K. , Toppari, J. , Slowikowska‐Hilczer, J. , Tuttelmann, F. , & Ferlin, A. (2020). European academy of andrology guidelines on Klinefelter syndrome. Journal of Andrology, 9, 145–167. 10.1111/andr.12909 32959490

[jgc470074-bib-0043] Zoom . (n.d.). Zoom (Version 5.16.10) [Software program] . https://zoom.us

