# Supplementary material for: Finding acceptance: How adolescents and young adults with Klinefelter Syndrome navigate identity, disclosure, and support
Source: J Genet Couns. 2025 Jun 24;34(3):e70074. doi: 10.1002/jgc4.70074 (PMC12186015; doi:10.1002/jgc4.70074)
Supplement: Supplementary file 1 — Data S1: [file JGC4-34-0-s001.docx]

**Experiences and Perspectives of Adolescents and Young Adults with Klinefelter Syndrome: Hormone Replacement, Future Fertility, and Psychosocial Well-Being**

**Qualitative Interview Guide**

1. *Introduce self, pronouns, and option to have video on/off*
2. *Thank them for participation (see script below)*
3. *Review e-consent if they have not already completed in advance*
4. *Review ground rules (see below)*
5. *Tell participant you are going to turn on the recording and that you will be stating the participant's ID# (ID # followed by interviewer initials: e.g., ID# 2 AT); Hit record and start by stating this information*
6. *Proceed with demographics and interview*

***Interview Script***

Thank you for speaking with me today. We’ve asked you to participate in this study because you are a teen or young adult with Klinefelter syndrome (XXY). We want to learn about your experiences because we think that this will help other people with Klinefelter syndrome (XXY). Before we get started, is there a term you prefer to use instead of Klinefelter syndrome such as XXY?

***Ground Rules***

Before we begin, I want to review 9 guidelines for the interview today:

1. I have never met you before, and I don't know much about you. I am hoping you can explain things to me-- you are the expert today.

2. You may not know the answer to some of the questions I ask you today. If you don't know an answer, that's ok--you should just tell me you don't know. Don't try to guess if you are unsure as there are no right or wrong answers, just your thoughts and experiences.

3. If I ask a question that you don't understand, please tell me. I can use different words. I might be accidentally confusing in the way I ask things sometimes. Always feel free to ask me questions or ask me to say something in a different way.

4. It is possible that I will ask a question that you don't want to answer. You don't have to answer any questions. I request that you don't make up an answer--you can just tell me that you don't want to answer that question.

5. It is possible that I may ask you a question more than once. Sometimes I forget things I've already asked. If I repeat a question it does not mean that there was anything wrong with your answer the first time.

6. If I say something wrong, please let me know. I don't always get everything right and I want to make sure that I understand you.

7. During the interview, I will be asking some questions about your medical team. When I refer to your medical team, this includes your doctors, nurses, and mental health providers like psychologists and social workers or anyone else involved in your medical care.

8. I am interested in your experiences and opinions. When the interview begins I will not be giving my opinion on what you say or your ideas, so that we can focus on your experiences. After the interview, if you have any questions for me about services, the project, or other things like that, I would be happy to answer them.

9. Finally, I will be audiotaping our interview today, and the interview will then be transcribed so that we can better understand and analyze the responses we receive from our participants. We will do our best to protect your confidentiality – for example, we will never associate your name with the information you share with me today in any publication resulting from the study. We may use a quote but we would never list your name or other information that would identify you along with that quote.

Do you have any questions?

***Demographic Questions***

Before we start, I would like to get to know you more, so I have some general questions about you:

- Because this interview will be audio recorded I will try not to use your name to maintain your privacy. If I do use your name I will be removing it manually from the transcript of the recording.
- I’m going to start the audio recording now.

***Begin recording now. State participant ID number and interviewer initials (e.g., ID#2, AT).***

- How old are you?
- How do you define or describe your gender?
- What pronoun(s) do you use?
- How do you define or describe your race or ethnicity?
- What grade/year in school are you in currently?
  - (*If not in school)* What is the highest level of education you’ve completed?
  - (*If currently in or graduated from college)* What is/was your major?
  - Are you currently working?
    - *If yes:* What do you do for work?
  - What area of work are you hoping to go into in the future? In other words, what is your dream job?
  - Tell me about how/when you were diagnosed with Klinfelter syndrome and when you found out about it?

*Identify the resources and support systems AYA with KS utilize to cope with the challenges of their diagnosis.*

I’m going to ask you some questions about people you get support from and other ways in which you may manage Klinefelter syndrome. Support people may include those who you feel comfortable talking to and getting help from and can include people like close friends, family members, romantic partners, teachers, coaches, instructors, etc.

- Can you tell me about who in your life you confide in or seek support from?
- What have you done to get support specific to having Klinefelter syndrome?
  - What was that like for you?
- How have you found information about Klinefelter syndrome, this can include places like the internet, books, or other people?
  - What has been most helpful to you in learning about Klinefelter syndrome?
- Can you tell me about any conversations about Klinefelter syndrome you had with your parents/caregivers?
  - Who initiated these conversations?
  - When did you talk about this?
  - How did you feel about these conversations?
  - What did you talk about?
- Have you told others about having Klinefelter syndrome? Who? (Repeat for each person they told)
  - How old were you when you told them?
  - What was it like for you to tell them?
  - What do you remember telling them?
  - How did they react to the information?
- What advice have you received about how to talk to other people about Klinefelter syndrome?
  - Who gave you the advice?
  - Tell me everything you remember about the advice you got.
- Has anyone encouraged or discouraged you from talking about Klinefelter syndrome? If yes, tell me more about that.
- Do you ever worry that negative things will happen if you share your Klinefelter syndrome with others? If so, can you tell me about those worries?
- What advice would you give other people with Klinefelter syndrome about sharing information, talking about it?
- Do you feel a sense of community with or feel connected to others with Klinefelter syndrome?
- Have you ever been a part of community organizations or support groups related to your Klinefelter syndrome?
  - *If yes…*
    - Tell me about those experiences.
    - When you interact with other people who have Klinefelter syndrome, what are the positive, or good things about that experience?
    - When you interact with other people who have Klinefelter syndrome, what are the negative, or bad things of that experience?
  - *If no…*
    - What are some of the reasons that keep you from being involved with these?

*Explore the psychosocial well-being of AYA with KS and how KS is perceived to impact it.*

I’m going to ask you some questions about school, work, relationships, body image, self confidence, and mental health.

- How does Klinefelter syndrome impact your school or your job?
  - What do you do to address these concerns?
  - What advice would you give to teachers/employers?
  - What advice would you give to healthcare providers?
- How has Klinefelter syndrome impacted your relationships with friends, romantic interests, or family?
  - What relationships do you feel have been impacted by Klinefelter syndrome?
  - How has Klinefelter syndrome impacted those relationships?
- Does having Klinefelter syndrome impact the way that you feel about the way you look or your appearance (often referred to as your body image)?
  - *If yes…*
    - How has Klinefelter syndrome impacted the way you feel about the way you look or your appearance?
- Are there ways Klinefelter syndrome has made your life better?
  - *If yes…*
    - How has Klinefelter syndrome made your life better?
- Are there ways having Klinefelter syndrome has made your life more difficult?
  - *If yes…*
    - How has Klinefelter syndrome made your life more difficult?
- How has the impact of Klinefelter syndrome on different areas of your life changed over time?
- Have you ever met with a mental health provider, such as a psychiatrist, psychologist, therapist or counselor?
  - *If yes…*
    - Can you tell me more about that?
      - Who did you see?
      - When did you start seeing them and for how long?
      - Why did you start seeing them?
    - *Have you talked to them about having Klinefelter syndrome? What was that like?
- Have you ever been prescribed or taken medication for an emotional or behavioral concern?
  - *If yes…*
    - Can you tell me more about that?
- What advice would you give to mental health providers who work with people that have Klinefelter syndrome?

*Explore the attitudes of AYA with KS about testicular dysfunction, specifically the likely need for testosterone replacement and, if desired, fertility-related interventions.*

I’m going to ask you about the different ways you have learned about Klinfelter syndrome and how you made decisions about your medical care.

- What is it like to see the endocrinologist (hormone doctor) every year and to have conversations about current and future medical needs?
- What is your understanding of why many people with Klinefelter syndrome take testosterone as a medication?
- Are you currently taking testosterone?
  - *If yes…*
    - Tell me about who was involved in making that decision to start testosterone?
    - Tell me about what type of testosterone you take and who was involved in making that decision? *(examples: injection every week, injection in the clinic every three months, gel applied to the skin, pellet injected under the skin (if an adult))*
    - How do you feel about having to take testosterone?
    - Do you feel motivated to regularly take your testosterone treatments?
      - *If yes…*
        - What motivates you to regularly take your testosterone?
    - What advice would you have for someone with Klinefelter syndrome who is about to start or just starting testosterone?
  - *If no…*
    - Have you talked about testosterone with doctors or your parents?
      - *If yes…*
        - What have those conversations been like?
- Have you talked about fertility, or being able to have kids?
  - *If yes…*
    - Who have you talked to about it with?
    - Tell me about those conversations and how you felt about them.
    - Were you given resources or information about fertility by your medical team?
    - Were you given the option to see a doctor specializing in fertility (i.e., a urologist)?
      - *If yes…*
        - Did you see the doctor specializing in fertility?

*If yes...*

Can you tell me more about what motivated you to see the fertility doctor?

Did you try to or give a semen sample at the doctor’s office?

What motivated you to give or try to give a semen sample

What do you recall about the results of your sample?

Did you learn about a surgical procedure (TESE) that can also be done to look for sperm?

What motivated you to consider TESE?

What do you want other AYA with Klinefelter syndrome to know about this experience?

What should they expect when they see the fertility doctor?

- - *If no…*
    - Have you thought about your fertility, or being able to have kids?
    - Do you want to talk to someone about your fertility?
      - *If yes…*
        - Who?
    - Do you see yourself ever talking to a doctor about your fertility?
- What advice would you give to medical professionals and families about sharing information related to fertility to others with Klinefelter syndrome?
- What advice would you give to doctors or other medical providers who will be working with children or young people who have Klinefelter syndrome?
  - How would your advice change based on the person’s age?
    - *Optional prompts…*
      - What advice would you give to doctors or other providers who will be working with young children who have Klinefelter syndrome?
      - What advice would you give to doctors or other providers who will be working with teens who have Klinefelter syndrome?
      - What advice would you give to doctors or other providers who will be working with young adults who have Klinefelter syndrome?
